# Supplementary material for: MetaRibo-Seq measures translation in microbiomes
Source: Nat Commun. 2020 Jun 29;11:3268. doi: 10.1038/s41467-020-17081-z (PMC7324362; doi:10.1038/s41467-020-17081-z)
Supplement: Supplementary file 10 — Supplementary Data 7 [file 41467_2020_17081_MOESM10_ESM.zip › File2/Confidence_VeryHigh_Taxonomy/332989_out.krona.html]

Javascript must be enabled to view this page.

members
magnitude
magnitudeUnassigned
count
unassigned
taxon
rank

332989\_out

11

superkingdom
11
2

11
976
phylum

class
11
200643

11
171549
order

11
171552
family

genus
838
11

2292054
5

SRS012849\_contig\_number\_19064SRS012969\_contig\_number\_17594SRS012989\_contig\_number\_contig-100\_24329.24329.24329SRS017307\_contig\_number\_contig-100\_2019.167748SRS077849\_contig\_number\_21890
species

species
1

SRS049959\_contig\_number\_21282
1262925

species
2293125

SRS053356\_contig\_number\_contig-100\_4432.241138
1

165179

SRS011134\_contig\_number\_7700
1
species

species
2292365

SRS023526\_contig\_number\_30939SRS049959\_contig\_number\_30372SRS077641\_contig\_number\_5528
3
